# The Effect of Azithromycin in Adults with Stable Neutrophilic COPD: A Double Blind Randomised, Placebo Controlled Trial

**DOI:** 10.1371/journal.pone.0105609

**Published:** 2014-08-22

**Authors:** Jodie L. Simpson, Heather Powell, Katherine J. Baines, David Milne, Harvey O. Coxson, Philip M. Hansbro, Peter G. Gibson

**Affiliations:** 1 Centre for Asthma and Respiratory Diseases and Hunter Medical Research Institute, Faculty of Health and Medicine, The University of Newcastle, Callaghan, New South Wales, Australia; 2 Department of Respiratory and Sleep Medicine, Hunter New England Area Health Service, Newcastle, New South Wales, Australia; 3 Auckland District Health Board, Auckland, New Zealand; 4 Department of Radiology and James Hogg Research Centre, University of British Columbia, Vancouver, Canada; University of Dundee, United Kingdom

## Abstract

**Background:**

Chronic Obstructive Pulmonary Disease (COPD) is a progressive airway disease characterised by neutrophilic airway inflammation or bronchitis. Neutrophilic bronchitis is associated with both bacterial colonisation and lung function decline and is common in exacerbations of COPD. Despite current available therapies to control inflammation, neutrophilic bronchitis remains common. This study tested the hypothesis that azithromycin treatment, as an add-on to standard medication, would significantly reduce airway neutrophil and neutrophils chemokine (CXCL8) levels, as well as bacterial load. We conducted a randomised, double-blind, placebo-controlled study in COPD participants with stable neutrophilic bronchitis.

**Methods:**

Eligible participants (n = 30) were randomised to azithromycin 250 mg daily or placebo for 12 weeks in addition to their standard respiratory medications. Sputum was induced at screening, randomisation and monthly for a 12 week treatment period and processed for differential cell counts, CXCL8 and neutrophil elastase assessment. Quantitative bacteriology was assessed in sputum samples at randomisation and the end of treatment visit. Severe exacerbations where symptoms increased requiring unscheduled treatment were recorded during the 12 week treatment period and for 14 weeks following treatment. A sub-group of participants underwent chest computed tomography scans (n = 15).

**Results:**

Nine participants with neutrophilic bronchitis had a potentially pathogenic bacteria isolated and the median total bacterial load of all participants was 5.22×10^7^ cfu/mL. Azithromycin treatment resulted in a non-significant reduction in sputum neutrophil proportion, CXCL8 levels and bacterial load. The mean severe exacerbation rate was 0.33 per person per 26 weeks in the azithromycin group compared to 0.93 exacerbations per person in the placebo group (incidence rate ratio (95%CI): 0.37 (0.11,1.21), p = 0.062). For participants who underwent chest CT scans, no alterations were observed.

**Conclusions:**

In stable COPD with neutrophilic bronchitis, add-on azithromycin therapy showed a trend to reduced severe exacerbations sputum neutrophils, CXCL8 levels and bacterial load. Future studies with a larger sample size are warranted.

**Trial Registration:**

Australian New Zealand Clinical Trials Registry ACTRN12609000259246

## Introduction

Chronic Obstructive Pulmonary Disease (COPD) is a major global health issue. Airway inflammation is recognised as a key element of COPD but its role in disease pathogenesis is poorly understood. Persistent neutrophilic airway inflammation (neutrophilic bronchitis) is a typical feature of COPD, which persists even after the removal of stimuli such as tobacco smoke. Neutrophil function in COPD is dysfunctional where clearance of antigens and micro-organisms is impaired and persistent activation contributes to further inflammation (neutrophil feedback cycle) and tissue destruction [1], [2].

The presence of neutrophilic bronchitis in COPD is linked to colonisation of the airways by bacteria and both airway neutrophils and the presence of colonising bacteria are associated with lung function decline [3], [4]. We have previously shown that in COPD, both sputum TLR2 gene expression and MMP9 protein levels were independent contributors to the proportion of neutrophils in sputum after correcting for age, smoking and airflow obstruction [5]. The clinical consequences of neutrophilic bronchitis include loss of lung function [6], however this feature remains largely untreated in COPD.

Macrolide antibiotics such as azithromycin (AZM) accumulate in host cells such as macrophages and neutrophils and have anti-inflammatory effects. These include the inhibition of inflammatory cytokines such as CXCL8 [7], reduced activation of neutrophils and enhanced phagocytosis of apoptotic neutrophils [8]. Macrolides are effective anti-inflammatory agents in different diseases. In cystic fibrosis, macrolides improve quality of life and prevent deterioration of lung function [9], in asthma they reduce sputum CXCL8 levels and improve quality of life [10] and in non-cystic fibrosis bronchiectasis and COPD they reduce exacerbations [11], [12].

In COPD, macrolide therapy has been largely used as a treatment for acute exacerbations [13]. Although the efficacy of macrolides in preventing exacerbations in COPD is undisputed, some participants experienced a large number of exacerbations despite taking azithromycin [13]. One explanation for the observed variability in the effectiveness of macrolides in preventing exacerbations in COPD is the heterogeneity of airway inflammation in affected patients. The reduction in exacerbations in COPD may be due to the suppression of neutrophilic inflammation and bacterial load in the airways. If this is the case, then targeting macrolides to COPD patients with neutrophilic bronchitis should give optimal efficacy and minimise side effects by reducing unnecessary exposure to therapy.

In this study we tested the hypothesis that azithromycin therapy would reduce CXCL8 levels, bacterial load and, consequently, neutrophilic inflammation in participants with neutrophilic COPD. To do this we identified participants with symptomatic COPD and stable neutrophilic bronchitis and determined the effect of the addition of oral azithromycin on the intensity and pattern of airway inflammation. This was achieved by measurement of total cell counts, cellular differential and cytokine levels present in induced sputum as well as alterations in bacterial load. We also assessed the rate of severe exacerbations where participants required unscheduled medical attention with treatment of oral corticosteroids and/or antibiotics over a period of 26 weeks from the baseline visit. We found that treatment reduced exacerbations but not neutrophilic bronchitis.

## Materials and Methods

The protocol for this trial and supporting CONSORT checklist are available as supporting information; see Checklist S1 and Protocol S1.

### Ethics statement

Participants gave written informed consent. The Hunter New England Area Health Service and University of Newcastle Research Human Ethics Committees approved the study (06/12/13/3.08 and H-2008-0272) and it was registered with the Australian New Zealand Clinical Trials Registry (ACTRN12609000259246).

### Participant recruitment

Recruitment targeted individuals who had symptomatic COPD with stable persistent neutrophilic bronchitis. Adults (males and non-pregnant females) who were more than 55 years of age with a doctors diagnosis of symptomatic COPD (n = 77) were recruited from a tertiary care setting at the Respiratory and Sleep Medicine Ambulatory Care Service, John Hunter Hospital, NSW, Australia. Inclusion criteria were a post bronchodilator FEV_1_/FVC <70% and FEV_1_<80% [14] and persistent neutrophilic bronchitis defined as sputum neutrophil proportion of more than 61% or more than 162×10^4^/mL sputum neutrophils demonstrated on two occasions (at least one being the screening visit). The neutrophil cut-off were based upon the upper limit of normal from healthy controls [15]. Participants had no reported exacerbations or alterations in respiratory medications in the previous 4 weeks. Additional exclusion criteria were the inability to produce an adequate sputum sample, a FEV_1_<0.5 L, current smoking or having ceased smoking in the past 6 months, a known hypersensitivity to macrolides, an ECG assessment showing a prolonged QTc interval ≥440 msec or an impairment of liver function.

**Figure 1:**
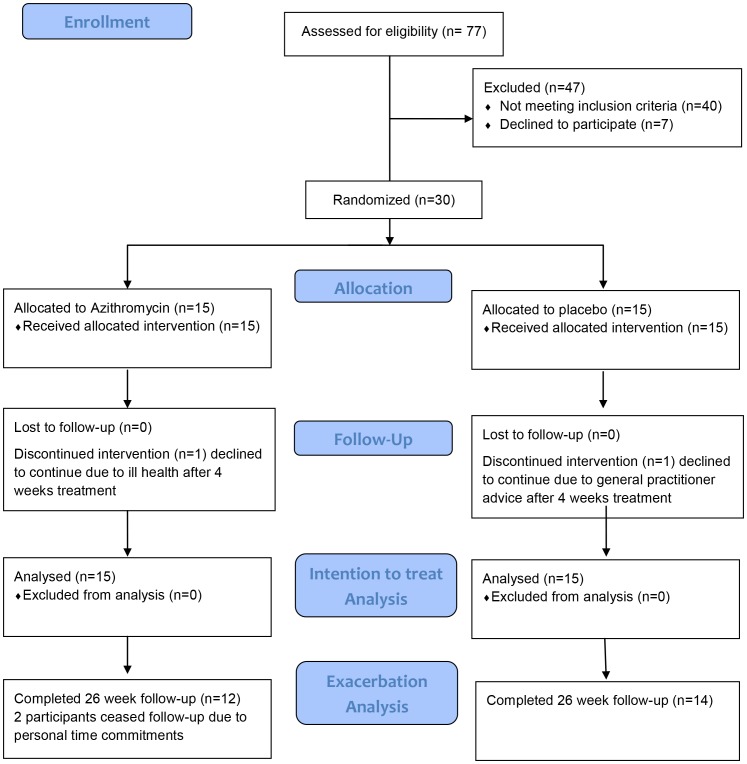
Participant consort diagram.

Following screening, 17 participants were excluded as they did not meet the lung function criteria, 16 did not meet the neutrophilic inflammation criteria, 3 were excluded as they did not meet the QTc or LFT criteria to enter the randomised controlled trial of azithromycin and 4 were unable to produce sufficient sputum. A further 7 participants declined to participate following screening (Figure 1).

Eligible participants (n = 30) were randomly allocated (1∶1) to receive oral azithromycin 250 mg daily or identical placebo for 12 weeks. Both participants and study staff were blinded to the assignment of intervention. In addition to the screening visit, randomised participants attended 4 visits at monthly intervals with the final study visit conducted 4 weeks after the end of treatment. Concealed random allocation was undertaken by a blinded staff member who took no further part in the study (HP). A random numbers table was computer generated (www.randomization.com) for treatment allocation using permuted blocks of six and participants were stratified according to smoking history (never or previous smokers). The active medication and placebo were prepared and packaged identically by a compounding chemist (Stenlake Science and Nature, Bondi Junction, NSW, Australia) and dispensed by the John Hunter Hospital pharmacy according to the random number table. After 12 weeks of treatment, participants were followed for an additional 12 weeks by monthly telephone interview and any changes in respiratory health recorded.

### Assessments

At screening (visit 1), pre and post bronchodilator spirometry, skin allergy prick testing, medication history, smoking status and exhaled carbon monoxide (eCO) were assessed and sputum induction was undertaken. Single breath diffusing capacity test was performed in those who had smoked cigarettes for more than 10 pack years (one pack year is approximated to 20 cigarettes daily for 1 year).

At visit 2 (baseline), mucus hypersecretion, St George Respiratory Questionnaire (SGRQ) [16], symptom visual analogue scores (VAS), Clinical COPD Questionnaire (CCQ) [17], modified Medical Research Council (mMRC) dyspnoea scale, sputum and blood were collected. Sputum was processed for inflammatory cell counts and supernatant stored for the assessment for inflammatory mediators.

A chest CT scan was performed in a subgroup of 15 participants as a secondary exploratory analysis.

#### Health care utilisation and quality of life

Health care utilisation was assessed by asking participants about their visits to hospital, emergency room and General Practitioner due to their chest disease and about courses of oral corticosteroids or antibiotics for their disease in the past 12 months. Quality of life was assessed using the SGRQ.

#### Mucus production and chronic bronchitis

Mucus production was noted as positive if the participant reported an affirmative answer to the following questions: ‘Do you cough and produce sputum/phlegm?’ or ‘Do you usually bring up phlegm from your chest?’, or ‘Do you usually have phlegm in your chest that is difficult to bring up when you don't have a cold?’. The presence of chronic bronchitis was assessed using questions taken from the American Thoracic Society and Division of Lung Diseases respiratory symptom questionnaire [18].

#### Smoking assessment

A smoking history was taken and smoking pack-years determined. Participants underwent eCO measurements, determined by electrochemical detection with a Smokerlyzer (Bedfont Scientific, Kent, UK; detection limit of 1 ppb). All included participants had an eCO of less than 10 ppm confirming their non-smoking status [19].

#### Severe exacerbations

A severe exacerbation was defined as an increase in symptoms requiring unscheduled medical attention and the use of oral corticosteroids and/or antibiotic treatment [20].

#### Pulmonary function tests

Three reproducible measurements of forced expiratory volume in 1 second (FEV_1_) and forced vital capacity (FVC) were obtained (KoKo, PD Instrumentation, Louisville, CO, USA) before and after inhalation of 200 µg salbutamol *via* a metered dose inhaler with valved holding chamber (Volumatic, Allen and Hanbury's, Australia). These measures were compared to predicted values according to Knudson *et al.*, [21]. The carbon monoxide transfer co-efficient (KCO) was determined according to ATS guidelines (MedGraphics Elite DX, Medical Graphics Corporation, St Paul, MN, USA) [22].

#### Chest computed tomography

CT scans were acquired using a multi-slice CT scanner (Cardiac 64 multi-slice scanner, Siemens, Forchheim, Germany) at the baseline visit and 4 weeks following the end of azithromycin treatment. The inspiratory and expiratory scans were measured using the CT analysis software Apollo (VIDA Diagnostics, Coralville, IA, USA) and the following parameters were obtained: total lung volume, mean lung density at both inspiration and expiration, the percentage of lung with densities below −950 Hounsfield units (HU) on inspiration (emphysema) and −856 HU on expiration (air trapping). The three-dimensional reconstruction of the airway tree was used to measure the airway lumen and wall area at the midpoint between airway junctions in all airways from the 3rd generation (segmental airways) to the 5th generation. Using these data the square root of wall area of all these bronchi were plotted against the internal perimeter for each subject. Individual regression equations were used to calculate the square root of the airway wall area for an idealized airway with an internal diameter of 10 mm. This parameter was chosen because it has been shown to predict the mean dimensions of histological small airways in COPD. Scans were also scored independently by a thoracic specialist radiologist (DGM) blinded to the subject group using a modified Bhalla scoring system as previously described [23].

#### Sputum induction, analysis and bacterial culture

Sputum was induced with hypertonic saline (4.5%) as previously described [24]. Selected sputum was dispersed using dithiothreitol (DTT). Sputum dispersed in DTT was diluted serially into skim milk powder, tryptone soya powder, glycerol and glucose (STGG) media [25] and chocolate bacitracin and blood agar plates were inoculated (10 µL) and incubated for 48 hours at 37°C, 5% CO_2_. Bacterial colonies were enumerated, cfu/mL calculated and sub-cultured for identification [3], [26]. Identification included Gram stain, oxidase and catalase testing, *Haemophilus* identification plates, staphylase and tributyrin tests.

A total cell count of leucocytes and viability was performed on filtered suspensions. Following centrifugation, supernatant was stored at −80°C. Cytospins were prepared, stained (May-Grunwald Geimsa) and a differential cell count obtained from 400 non-squamous cells. Supernatants were stored for the assessment of CXCL8 and neutrophil elastase (NE). CXCL8 was assessed using a commercial ELISA kit (R&D Systems, Minneapolis, MN, USA) and NE was measured with the InnoZyme Human Neutrophil Elastase Immunocapture Activity Assay (Calbiochem, Merck, Kilsyth, Victoria, Australia).

#### Adverse events

Adverse events including the presence of fever, headache, nausea, vomiting, diarrheoa and skin rashes were recorded at each study visit and at fortnightly intervals between study visits.

#### Statistical methods

Statistical analysis was performed using Stata 11 (StataCorp, College Station, TX, USA). Results are presented as mean ± standard deviation (SD) or median (interquartile range (IQR)) with Student's t-test for parametric data and Wilcoxon rank-sum tests for nonparametric data. Paired data were analysed using the Wilcoxon signed-rank test. Categorical data were compared using the Chi-square or Fisher's exact test as appropriate. Two-sided tests with p values <0·05 were considered significant. The primary outcome variable of this study was a reduction in sputum CXCL8; secondary outcome variables were change in sputum neutrophil proportion and total bacterial load. This study was powered to detect a change of 250 pg/mL in CXCL8 based on a previous randomised controlled trial of macrolide therapy in COPD [27]. Exacerbation data were analysed on an intention-to-treat basis. A Poisson regression model was used to compare the exacerbation rate difference between the treatment groups. The time to first exacerbation was analysed using Kaplan-Meier plots and analysis of instantaneous risk described using a Cox proportional hazards model. Analysis of covariance was conducted on end of study QoL and sputum variables with adjustment for baseline values. Other continuous variables such as symptoms were analysed using a generalised linear mixed model with a random intercept for individuals to account for the repeated measurements on individuals.

## Results

### Patient participation

Seventy-seven patients were screened for the study between April 2009 and December 2011. Thirty eligible participants (19 male, Figure 1) underwent detailed clinical and inflammatory assessment and subsequent random allocation to treatment (15 to azithromycin and 15 to placebo). Twenty eight entered the follow-up period, 2 did not complete the 12 week treatment as they were excluded (Figure 1). Analyses of the effects of treatment were performed on 30 participants (19 male) who completed the study (Table 1).

**Table 1:** Participant characteristics.

	All participants	Placebo	Azithromycin	p value
N	30	15	15	
**Clinical parameters**				
Age, mean (SD); range	70.8 (7.6)	69.9 (8.9)	71.7 (6.2)	0.535
Sex, Male/Female	19/11	10/5	9/6	0.705
Ex-smokers, n (%)	22 (73.3%)	11 (73.3)	11 (73.3)	1.0
Pack years, mean (SD)	46.11 (36.61)	56.2 (43.2)	36.0 (26.9)	0.202
FEV_1_% predicted, mean (SD)	53.69 (13.74)	51.1 (13.7)	56.5 (13.7)	0.297
FEV_1_/FVC, mean (SD)	57.79 (11.24)	51.3 (11.3)	52.3 (11.6)	0.811
Atopy, n (%)	14 (46.67%)	8 (53.3)	6 (40.0)	0.464
ICS dose, BDP equivalent, µg/day, mean (range)	1011, (400–2000)	800 (500–1000), N = 15	1000 (800–2000), N = 11	0.196
CCQ total score, mean (SD)	16.0 (17.6)	16.5 (6.97)	15.4 (8.47)	0.692
SGRQ total score, mean (SD)	34.2 (16.0)	33.8 (15.7)	34.5 (16.8)	0.907
mMRC dyspnea score, mean (SD)	0.90 (0.80)	0.87 (0.92)	0.93 (0.70)	0.825
**Inflammatory outcomes**				
Total cell count, ×10^6^/mL, median (q1,q3)	5.54 (3.78,9.54)	5.58 (3.78,9.54)	4.68 (3.33,10.71)	0.604
Viability, %, median (q1,q3)	88.0 (78.5,92.5)	87.12 (73.9,92.86)	88.4 (78.99,92.5)	0.788
Neutrophils, %, median (q1,q3)	65.63 (46.5,71.25)	68 (46.5,82.3)	63.5 (42.75,71.0)	0.547
Neutrophils, ×10^4^/mL, median (q1,q3)	368.9 (209.3,556.9)	380.4 (209.3,556.9)	251.5 (165.4,570.7)	0.468
Eosinophils, %, median (q1,q3)	2.03 (1.25,3.50)	1.75 (1.25,3.0)	3.25 (1.25,8.0)	0.140
Eosinophils, ×10^4^/mL, median (q1,q3)	10.85 (5.74,30.07)	10.2 (4.3,18.5)	16.1 (5.7,69.0)	0.468
Macrophages, %, median (q1,q3)	31.63 (19.0,43.75)	30.25 (15.1,46.5)	32.5 (21.5,43.75)	0.885
Macrophages, ×10^4^/mL, median (q1,q3)	171.7 (117.0,248.0)	209.3 (140.0,289.7)	130.7 (88.7,205.7)	0.152
Lymphocytes, %, median (q1,q3)	0.25 (0,1.25)	0.25 (0,1.75)	0.5 (0,0.75)	0.470
Lymphocytes, ×10^4^/mL, median (q1,q3)	2.57 (0,6.46)	3.7 (0,9.0)	1.2 (0,5.7)	0.197
Columnar epithelials, %, median (q1,q3)	0.5 (0,1.75)	0.5 (0,2.25)	0.5 (0,1.25)	0.525
Columnar epithelials, ×10^4^/mL, median (q1,q3)	2.30 (0,5.36)	2.5 (0,13.5)	1.6 (0,5.0)	0.460
Squamous, %, median (q1,q3)	16.33 (9.17,41.73)	5.88 (0.74,11.31)	3.14 (1.48,6.54)	0.694
CXCL8, ng/mL, median (q1,q3)	16.22 (8.50,32.93)	25.37 (10.32,58.88)	11.93 (6.19,21.93)	0.120
NE, ng/mL, median (q1,q3)	3038 (1318,6872)	3868 (1722,10272)	1950 (901.3,4756)	0.141
**Bacteriology**	N = 26	N = 14	N = 14	
Bacterial load, ×10^7^ cfu/mL, median (q1,q3)	7.01 (1.84,14.5)	6.55 (1.84,14.00)	5.02 (1.65,20.0)	0.783
Bacterial pathogen isolated, n (%)	9 (37%)	3 (23%)	6 (46%)	0.205
**Computed tomography**				
Quantitative scores	N = 14	N = 7	N = 7	
Inspiratory lung density, mean (SD)	−856.82 (22.07)	−844.55 (23.14)	−869.08 (12.99)	0.031
% below −950 HU, inspiratory, mean (SD)	14.53 (9.86)	11.96 (9.66)	17.09 (10.10)	0.351
% below −856 HU, inspiratory, mean (SD)	64.73 (8.90)	58.95 (8.81)	70.52 (3.97)	0.008
Expiratory/Inspiratory mean lung density, mean (SD)	0.92 (0.04)	0.92 (0.05)	0.93 (0.03)	0.536
Inspiratory PI, mm, mean (SD)	3.82 (0.07)	3.84 (0.10)	3.80 (0.05)	0.459
Expiratory PI, mm, mean (SD)	3.94 (0.12)	3.94 (0.17)	3.95 (0.09)	0.902
Qualitative scores	N = 17	N = 9	N = 6	
Extent of bronchiectasis score, median (q1,q3)	1.0 (0.0,5.5)	5 (1.0,8.0)	0.5 (0.0,2.0)	0.160
Severity of bronchiectasis score, median (q1,q3)	1.0 (0.0,3.5)	3.0 (1.0,5.0)	0.5 (0.0,2.0)	0.160
Bronchial wall thickness score, median (q1,q3)	3.0 (2.0,4.5)	3 (3,5)	2 (0,4)	0.204
Bronchial wall thickness >2, n (%)	2.0 (12.0%)	2 (20%)	0 (0%)	0.331
Total lung score, median (q1,q3)	5.5 (4.0,12.0)	9 (4,16)	5 (0,6)	0.102
Total emphysema score, median (q1,q3)	0.0 (0.0,20.0)	2.1 (0.0,23.3)	0.0 (0.0,20.0)	0.673
Number of lobes decreased attenuation >0, median (q1,q3)	5.0 (3.0,6.0)	5 (4,6)	3 (2,5)	0.088
Mucus plugging (large or small airways), n (%)	2.0 (12.0%)	2 (20%)	0 (0%)	0.331
Consolidation present in any lobe, n (%)	5.0 (29.4%)	2 (20%)	3 (43%)	0.314

BDP equivalent: dose of inhaled corticosteroids is calculated as beclomethasone equivalents where 1 µg of beclomethasone  = 1 µg budesonide  = 0.5 µg fluticasone; CCQ: Clinical COPD Questionnaire; SGRQ: St George Respiratory Questionnaire; mMRC: modified Medical Research Council; cfu: colony-forming units; PI: Internal perimeter.

The included participants had a mean age of 71 years and 22 (73%) had smoked previously with a median of 46 pack years. Eleven participants had a smoking history of more than 10 pack years and this group had a mean (q1,q3) KCO% predicted of 62 (49,84). Nineteen participants (63%) had airflow limitation of moderate severity according to the Global initiative for chronic Obstructive Lung Disease (GOLD) guidelines [28]. Using the most recent descriptors for GOLD severity categories which incorporate the mMRC score and exacerbation history, 21 (70%) participants with neutrophilic COPD were categorised as high risk (Quadrant C-17 participants and Quadrant D-4 participants), with the remaining 9 (30%) participants were designated as low risk, 7 with low symptoms and 2 with high symptoms (Quadrant A and B respectively) [28]. At each visit a sputum sample was attempted for each of the 30 participants; of the 150 attempts, 135 had a sputum sample collected (90%) and an adequate sputum sample was produced on 122 occasions (90%).

Participants had significant neutrophilia (median 66% sputum neutrophils). At randomisation, 25 participants had culture results, 9 with potentially pathogenic bacteria. The most common bacteria cultured was *Pseudomonas aeruginosa* (n = 4) and *Streptococcus pneumoniae* (n = 2), *Haemophilus influenzae* (n = 1), *Moraxella cattarhalis* (n = 1) and *Staphylococcus aurerus* (n = 1).

### Response to azithromycin

**Figure 2:**
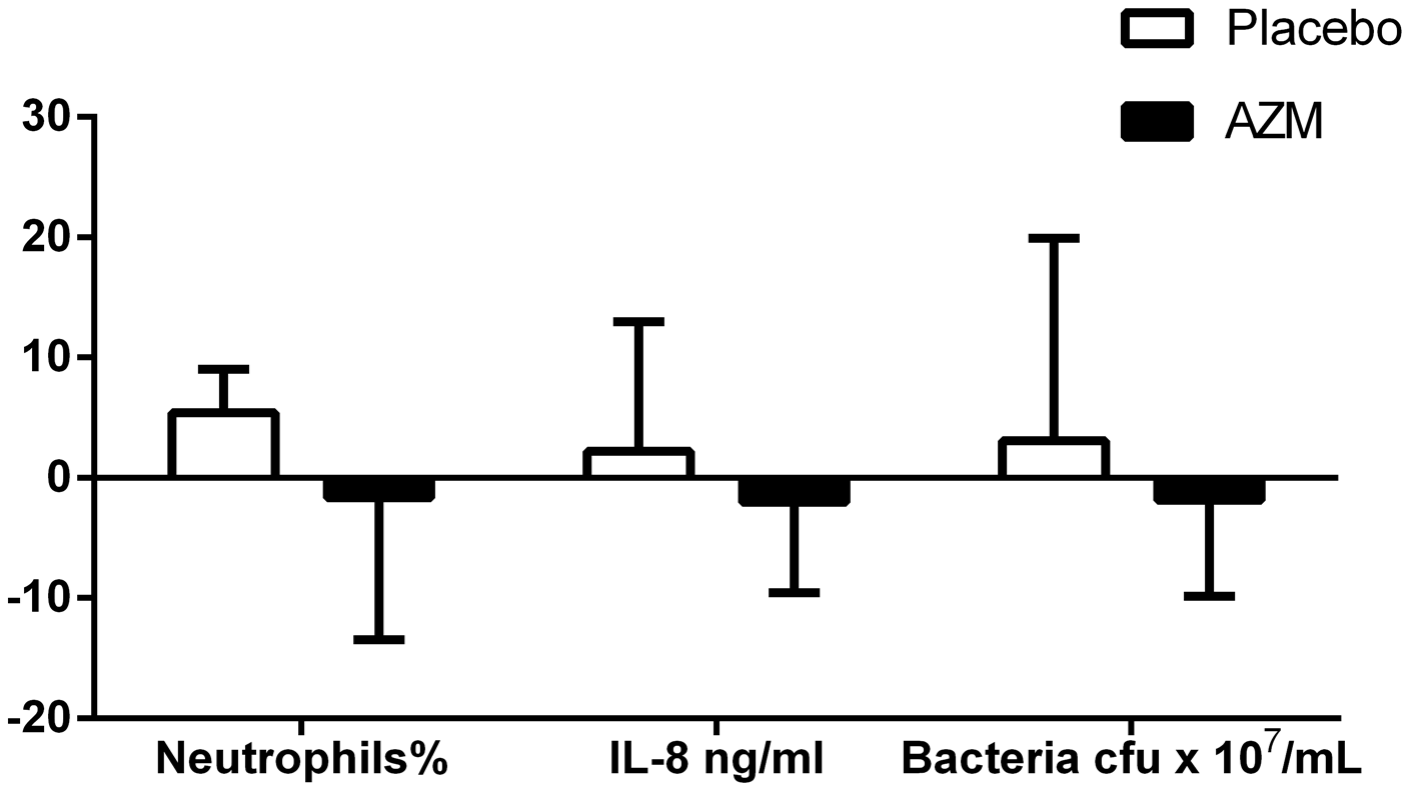
Bar graphs showing the change in sputum neutrophil%, IL-8 ng/mL and total bacterial load following treatment with azithromycin (AZM) or placebo.

There were no significant differences between treatment groups at baseline with respect to age, gender, atopy, lung function, quality of life, or daily inhaled corticosteroid (ICS) dose (Table 1). At baseline there were significantly more gas trapping in those treated with azithromycin compared to placebo (Table 1). There was a non-significant reduction in sputum neutrophil proportion, levels of CXCL8 and airway bacterial load in those who received add-on azithromycin compared to placebo shown in Figure 2. There were no differences in symptom score, SGRC, CCQ, lung function and CT scores at the end of treatment comparing azithromycin to placebo despite correction for baseline variation (Table 2).

**Table 2:** End of treatment clinical and inflammatory outcomes.

	Placebo	Azithromycin	p value	p value
**Clinical outcomes**		N = 15		
FEV_1_% predicted, mean (SD)	52.17 (14.3)	57.79 (13.90)	0.285	0.748¥
FEV_1_/FVC, mean (SD)	50.19 (10.33)	54.70 (13.03)	0.409	0.136¥
CCQ total score, mean (SD)	15.1 (9.2)	16.9 (10.1)	0.614	0.329#
SGRQ total score, mean (SD)	28.1 (13.2)	34.2 (15.9)	0.259	0.098#
mMRC dyspnea score, median (q1,q3)	1 (0,1)	1 (0,2)	0.695	1.000#
VAS Breathlessness, median (q1,q3)	27 (0,43)	27 (7,68)	0.676	0.101#
VAS Wheeze, median (q1,q3)	2 (0,31)	2 (0,28)	0.829	0.751#
VAS Cough, median (q1,q3)	18 (8,42)	14 (0,63)	0.868	0.380#
VAS Chest tightness, median (q1,q3)	5 (0,31)	8 (0,31)	0.542	0.937#
**Inflammatory outcomes**	N = 15	N = 15		
Total cell count, ×10^6^/mL, median (q1,q3)	4.32 (2.25,7.38)	3.96 (1.89,8.73)	0.787	0.862#
Viability, %, median (q1,q3)	77.5 (75.0,91.29)	85.7 (63.8,94.03)	0.663	0.946#
Neutrophils, %, median (q1,q3)	60.0 (42.25,82.75)	61.63 (56.5,78.5)	0.627	0.695#
Neutrophils, ×10^4^/mL, median (q1,q3)	372.4 (223.7,459.0)	186.8 (122.7,470.9)	0.577	0.578#
Eosinophils, %, median (q1,q3)	1.25 (1.0,2.5)	3.0 (0.5,7.25)	0.527	0.466#
Eosinophils, ×10^4^/mL, median (q1,q3)	7.88 (3.83,15.98)	10.52 (1.62,31.68)	0.884	0.251#
Macrophages, %, median (q1,q3)	34.75 (12.75,41.4)	32.25 (19.0,38.75)	1.000	0.810#
Macrophages, ×10^4^/mL, median (q1,q3)	109.15 (76.68,225.68)	99.14 (65.61,227.12)	0.734	0.897#
Lymphocytes, %, median (q1,q3)	0.5 (0.25,1.0)	0.25 (0,0.5)	0.156	0.113#
Lymphocytes, ×10^4^/mL, median (q1,q3)	2.16 (1.13,6.68)	0.64 (0,4.10)	0.125	0.339#
Columnar epithelial cells, %, median (q1,q3)	1.2 (0,2.25)	0.25 (0,1.5)	0.416	0.887#
Columnar epithelial cells, ×10^4^/mL, median (q1,q3)	6.38 (0,10.48)	1.62 (0,4.32)	0.523	0.690#
CXCL8, ng/mL, median (q1,q3)	33.0 (5.95,57.38)	9.22 (4.97,41.03)	0.310	0.825#
NE, ng/mL, median (q1,q3)	3977.4 (435.8,8182.1)	1496.2 (564.3,5408.9)	0.468	0.470#
**Bacteriology**	N = 13	N = 13		
Bacterial load, ×10^7^ cfu/mL, median (q1,q3)	5.10 (3.29,33.0)	2.37 (1.65,15.5)	0.285	0.755#
Pathogen isolated, n (%)	3 (23%)	5 (35%)	0.336	
**Computed tomography**				
Quantitative scores	N = 5	N = 6		
Inspiratory lung density, mean (SD)	−853.52 (30.30)	−865.75 (17.30)	0.403	0.966#
% below −950 HU, inspiratory, mean (SD)	15.51 (11.55)	14.81 (8.37)	0.904	0.947#
% below −856 HU, inspiratory, mean (SD)	62.19 (12.60)	69.52 (7.98)	0.246	0.891#
Expiratory/Inspiratory mean lung density, mean (SD)	0.92 (0.05)	0.92 (0.02)	0.938	0.902#
Inspiratory PI, mm, mean (SD)	3.84 (0.05)	3.83 (0.04)	0.698	0.875#
Expiratory PI, mm, mean (SD)	3.93 (0.17)	3.91 (0.10)	0.797	0.224#
Qualitative scores	N = 9	N = 5		
Extent of bronchiectasis score, median (q1,q3)	5 (1,8)	0 (0,1)	0.086	0.971#
Severity of bronchiectasis score, median (q1,q3)	3 (1,5)	0 (0,1)	0.086	0.775#
Bronchial wall thickness score, median (q1,q3)	4 (3,5)	1 (0,30)	0.092	0.793#
Bronchial wall thickness score >2, n (%)	2 (100%)	0 (0%)	0.486	
Total lung score, median (q1,q3)	11 (6,17)	5 (0,5)	0.038	0.754#
Total emphysema score, median (q1,q3)	0 (0,23.33)	0 (0,6.67)	0.425	0.696#
Number of lobes decreased attenuation >0, median (q1,q3)	5 (4,6)	3.5 (3,4)	0.114	0.599#
Mucus plugging (large or small airways), n (%)	3 (75%)	1 (25%)	0.604	
Consolidation present in any lobe, n (%)	2 (40%)	3 (60%)	0.329	

¥ GLMM: Generalised Linear Mixed Model;

# ANCOVA: adjusted for baseline and robust option for non-parametric data; CCQ: Clinical COPD Questionnaire; SGRQ: St George Respiratory Questionnaire; mMRC: modified Medical Research Council; VAS: visual analogue scale; cfu: colony-forming units; PI: Internal perimeter.

Twenty-two participants had samples for bacterial culture at randomisation and end of treatment visits, of which 10 had no identifiable pathogen at both visits (43%). Three participants had *Pseudomonas aeruginosa* identified at both visits (2 from the AZM and 1 from the Placebo group). Of the remaining participants with paired culture results, 5/9 were culture positive at randomisation (3 AMZ, 2 placebo) and negative at the end of the study, 4/9 were culture negative at randomisation and culture positive at the end of treatment (2 AZM and 2 placebo).

The total bacterial load was reduced by 53% following AZM treatment from 5.02×10^7^ cfu/mL to 2.37×10^7^ cfu/mL. In the placebo group there was also a reduction in total bacterial load of 37% from a median of 6.55×10^7^ cfu/mL to 4.14×10^7^ cfu/mL. We assessed the bacterial load of all bacteria identifiable in the samples and found similar total bacterial loads as to those reported by Wilkinson *et al*. [29]. We then selected a cut point of >10^8^ cfu/mL to define those patients who had a high bacterial load and found there were 10 participants with high bacterial load who had paired data pre and post treatment (5 AZM and 5 placebo). There was no difference in neutrophil proportion or CXCL8 levels following add-on azithromycin treatment in those with a high bacterial load. The bacterial load did reduce by 10 fold but this was not statistically significant, shown in Table 3.

**Table 3:** Sputum neutrophil proportion, CXCL8 levels and bacterial load before and following add-on azithromycin therapy in those participants with a high bacterial load (>108 cfu/mL) at baseline.

	Visit 2	Visit 5	p value
	N = 5	N = 5	
Sputum neutrophil, %	71.0 (60.75,75.0)	77.25 (57.5,78.5)	0.893
CXCL8, ng/mL	26.62 (21.94,34.99)	25.42 (24.82,41.03)	0.686
Bacterial load, ×10^7^ cfu/mL	21.9 (20,22)	1.79 (1.65,10.1)	0.225

**Figure 3:**
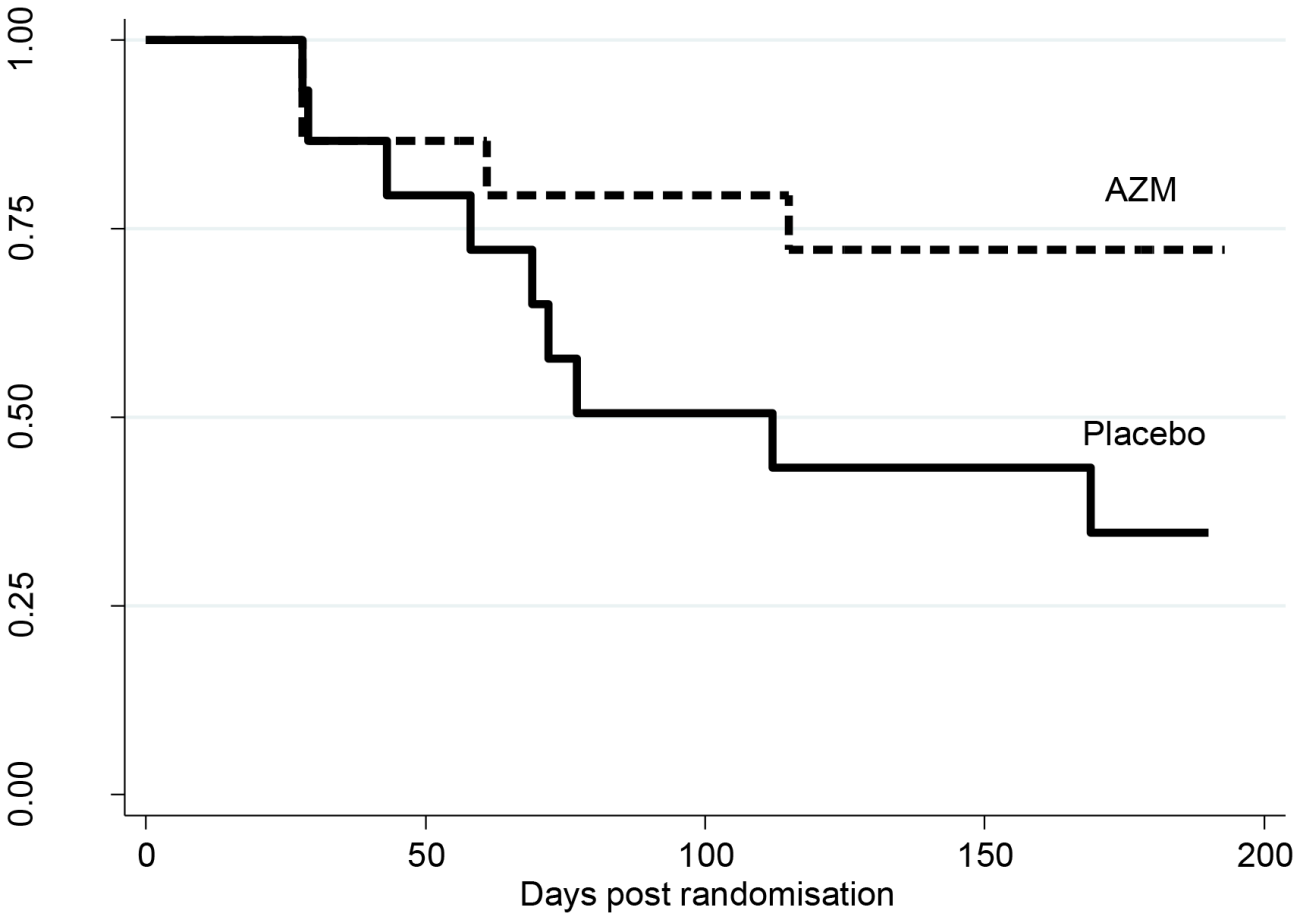
Kaplan-Meier curves showing the proportion of participants without a COPD exacerbation versus the days post randomisation visit (p = 0.100).

There was a clinically significant reduction in the median number of severe exacerbations experience by those treated with add-on azithromycin compared to placebo. With azithromycin, the median (IQR) number of severe exacerbations experienced was 0 (0,1), which was significantly less than with placebo (1 (0,2); p = 0.046). The mean severe exacerbation rate was 0.33 exacerbations per person per 26 weeks in the azithromycin group compared to 0.93 exacerbations per person in the placebo group (incidence rate ratio (95%CI): 0.38 (0.14,1.05, p = 0.062)). Fewer participants in the azithromycin group experienced a severe exacerbation, 4 (26.7%) compared to 9 (60%) in the placebo group (p = 0.139). The azithromycin group were 63% less likely to exacerbate at any time point (hazards ratio (95%CI): 0.37 (0.11,1.21), p = 0.100, Figure 3).

The most common macrolide-related side effect reported was diarrhoea in 5 participants taking azithromycin and 1 participant taking placebo. In all cases the side effect did not cause the participant to cease study medication or withdraw from the study (Table 4).

**Table 4:** Adverse events during treatment.

	Placebo N = 15	Azithromycin N = 15	p value
Diarrhoea	5 (33%)	1 (7%)	0.169
Abdominal pain	0 (0%)	0 (0%)	
Nausea	0 (0%)	0 (0%)	
Vomiting	0 (0%)	0 (0%)	
Fever	0 (0%)	0 (0%)	
Headache	0 (0%)	0 (0%)	
Rash	0 (0%)	0 (0%)	
Hearing loss	0 (0%)	0 (0%)	
Other	9 (60%)	10 (67%)	0.704

n (%), Fisher's exact test.

## Discussion

Participants with symptomatic COPD and stable neutrophilic bronchitis exhibited typical features of COPD including a previous history of cigarette smoking, presence of potentially pathogenic bacteria and health care utilisation for exacerbations. Twelve weeks of add-on azithromycin resulted in a clinically significant reduction in severe exacerbations and a non-significant reduction in neutrophilic airway inflammation, sputum CXCL8 levels and bacterial load.

These findings suggest that the exacerbation-reducing effects of macrolides in COPD shown in a recent meta-analysis [30] may not due to reductions in neutrophilic inflammation or bacterial load, as seen in individuals with cystic fibrosis and diffuse panbronchiolitis. This kind of heterogeneity in the response to macrolides is similar to that observed in participants with diffuse panbronchiolitis and small airways disease, where clinical improvements were observed in both groups but only those with diffuse panbronchiolitis had reductions in neutrophils [31]. The study of Albert and colleagues elegantly show the improvement in exacerbation rate and health status in COPD, however they did not assess changes in airway inflammation. Interestingly, the *post-hoc* analysis from the largest randomised controlled trial of macrolides in COPD reported participants with least severe disease and those who were not taking inhaled corticosteroids, macrolides had a more favourable effect in reducing exacerbations[13], suggesting that in COPD macrolides may be most effective in those with more mild disease. In this study, most participants had moderately severe COPD and 90% were taking inhaled corticosteroids.

The lack of significance observed in the inflammatory outcomes may be due to insufficient numbers of patients randomised. We assessed the possibility of a type 2 error to explain the lack of significant changes in sputum neutrophils with azithromycin treatment and found there was sufficient power (0.819) to detect an effect of azithromycin on neutrophils if one were present. A previous trial of azithromycin for non-eosinophilic/neutrophilic airways disease [32] found a reduction in neutrophils of 0.597×10^6^ neutrophils/mL. Our trial had a power of 82% (alpha 0.05) to detect this effect. Since an effect was not observed, it suggests azithromycin reduces severe exacerbations in neutrophilic COPD by other means. Alternative mechanisms may include prevention of bacterial infection-induced exacerbations, reductions in other inflammatory responses [33], or a synergistic symptom-controlling mechanism with other respiratory medications that is not through reductions in inflammation.

In previous studies, addition of clarithromycin to inhaled corticosteroid therapy resulted in a small but significant reduction in neutrophil proportion but no change in neutrophil number or of CXCL8 levels [34]. Other studies showed a significant reduction the number of neutrophils and levels of NE using erythromycin three times daily at both 3 and 6 month treatment end points [35] and a 12 month treatment study with twice daily erythromycin failed to reduce neutrophil numbers or CXCL8 levels [36].

An alternative explanation for the lack of reduction in neutrophilic inflammation in our study could be the type of macrolide used. We selected azithromycin due to its long half-life, fewer side effects and once daily dosing as particularly suitable for patients with COPD. A recent study of the efficacy of macrolide antibiotics to inhibit inflammatory cytokine production by COPD sputum cells showed that clarithromycin and roxithromycin were more potent than azithromycin [37]. This may explain why the two studies that used clarithromycin in COPD showed reductions in neutrophils [34], [38].

This study also assessed high resolution chest computed tomography scans in a sub-group of 17 participants, of which 14 had paired data before and after azithromycin add-on therapy. At baseline there appeared to be more gas trapping in the group that went on to receive treatment with azithromycin compared to the group who was to receive the placebo treatment. However at the end of treatment after correcting for baseline data there was no difference in any of the CT scores. As this analysis was only performed on a small number of participants', further research is necessary to determine if add-on azithromycin therapy in COPD alters gas trapping and other airway scores.

In conclusion, we have characterised and investigated add-on azithromycin therapy in participants with neutrophilic COPD. Add-on azithromycin therapy demonstrated a trend for a reduction in the number of severe exacerbations experienced and in the markers of neutrophilic airway inflammation. This study has not identified the mechanism by which azithromycin leads to reduced exacerbations, however, it suggests that the anti-exacerbation effect of add-on azithromycin in COPD may not be through an anti-neutrophilic mechanism. Further work is needed to determine the mechanism that leads to reduced exacerbations and a study with a larger sample size is warranted and can be designed using the data from this report.

## Supporting Information

Protocol S1 **Trial protocol.**
(PDF)

Checklist S1 **CONSORT checklist.**
(PDF)
