# Supplementary material for: The Effect of Azithromycin in Adults with Stable Neutrophilic COPD: A Double Blind Randomised, Placebo Controlled Trial
Source: PLoS One. 2014 Aug 22;9(8):e105609. doi: 10.1371/journal.pone.0105609 (PMC4141795; doi:10.1371/journal.pone.0105609)
Supplement: Protocol S1 — Trial protocol. (PDF) [file pone.0105609.s001.pdf]

A double-blind randomised controlled study of the anti-inflammatory effects of azithromycin 250mg, daily for 12 weeks in adults with symptomatic neutrophilic airway disease

Chief Investigator: Dr Jodie L Simpson
Post Doctoral Fellow

Co-Investigators: Professor Peter G Gibson
Associate Professor Philip Hansbro
Dr Katherine Baines

Address: Department of Respiratory Medicine
John Hunter Hospital
Locked Bag 1
Hunter Region mail Centre
NSW 2310
Australia

Introduction

Obstructive Airway Diseases (OADs) such as asthma and COPD are now major health issues for Australia. Airway inflammation is recognised as a key element of airway disease, but its role is poorly understood. Persistent neutrophilic airway inflammation (neutrophilic bronchitis) is considered a typical feature of COPD and we have recently described a subgroup of patients with asthma who also have neutrophilic inflammation. Each of these conditions is associated with airflow obstruction. Importantly, we found that the mechanisms underlying the development of airway neutrophilia were similar and remarkably consistent across the different conditions. We found that the induction of neutrophilic bronchitis involved upregulation of gene expression and protein secretion of the potent neutrophil chemoattractant IL-8 (CXCL8) (1), as well as neutrophil protease release.

There is a need to identify effective treatment options for people with OAD and neutrophilic inflammation. The anti-inflammatory effects of macrolides include the inhibition of inflammatory cytokines such as IL-8 (4), reduced activation of neutrophils and enhanced phagocytosis of apoptotic neutrophils (5). The discovery of these anti-inflammatory properties *in vitro* led to the investigation of macrolide antibiotics in the treatment of airway diseases such as diffuse panbronchiolitis (6) where similar reductions in neutrophils and IL-8 levels were observed. Macrolides have also been shown to lower IL-8 levels in chronic sinusitis (7) and are effective anti-inflammatory agents in cystic fibrosis showing improvements in quality of life and prevention of the deterioration of lung function (8). We have recently shown that macrolide antibiotic use in asthma can reduce IL-8 levels and improve quality of life (9).

Macrolide antibiotics such as azithromycin (AZM) are concentrated in host cells such as macrophages and neutrophils and have been shown to have anti-inflammatory effects. Azithromycin is a 15-membered ring macrolide derivative of erythromycin. It has a number of advantages over erythromycin including fewer side effects ands also the possibility of once daily dosing due to its long elimination half-life.

We propose to conduct a randomized double blind placebo controlled treatment trial in participants with neutrophilic airway disease (asthma or COPD) to investigate the anti-

inflammatory effect of AZM on airway inflammation. The primary outcome of this trial will be reduced levels of IL-8 in sputum, however other outcomes will also be assessed such as neutrophil numbers (%), sputum bacterial load (colony forming units/mL), levels of neutrophil elastase. Clinical outcomes will also be determined including symptoms, lung function and quality of life (St George's Respiratory Questionnaire) and severe exacerbations. Participants will be recruited to attend a screening visit, and if stable, they will enter a 2-week run-in period and then be randomised to either AZM (250mg) or placebo daily for twelve weeks. Study visits will occur at monthly intervals where symptoms, lung function and induced sputum will be assessed. A post treatment visit will be conducted 4 weeks after the end of treatment and then we will contact participants by telephone to monitor their airways disease and see if they have had an increase in their symptoms or medication use (exacerbation). We will monitor patients symptoms and medication use for a total of 6 months from their enrolment in the study.

Sample size - In a recent study, the effect of clarithromycin in COPD (10) was found to reduce sputum IL-8 levels from 1606 (367) to 882 (144) pg/mL. We will recruit 40 participants (this includes a 10% withdrawal rate) which will give sufficient power (80%) to detect a change in IL-8 levels in the order of 250pg/mL (alpha 0.05).

Study Objectives

To measure the effect of the addition of oral azithromycin treatment in participants with neutrophilic airway disease

1. The intensity and pattern of airway inflammation as assessed by total cell count, cellular differential and cytokine levels present in induced sputum.
2. Clinical status as measured by symptom scores, quality of life measures and pulmonary function tests.
3. Alteration in bacterial load

To determine the rate and inflammatory pattern of exacerbations in participants with neutrophilic airways disease over 6 months

Hypothesis

AZM therapy will reduce interleukin (IL)-8 levels, bacterial load and consequently neutrophilic inflammation in participants with neutrophilic airway disease.

Study Design

This is a double blind placebo controlled study. This study will last for 6 months and involve a minimum of 6 visits to the research unit and telephone consultations.

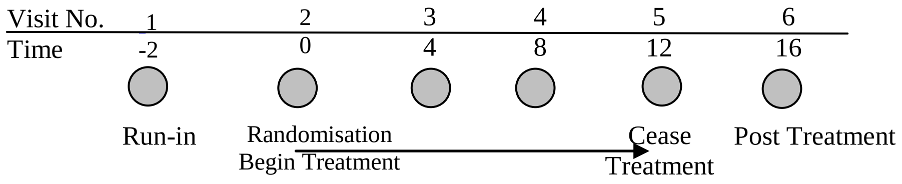

Participant Selection

Inclusion Criteria

- Diagnosis of symptomatic airway disease (asthma or COPD)
- Male or Female over the age of 55 years
- No change in treatments for respiratory disease for 1 month prior to randomisation
- Informed consent obtained
- Increased neutrophils (>61% or >162 x10^4 /mL) on two occasions (at least one during run-in)
- FEV_1<FVC ratio <70% AND FEV_1% predicted <80%
- Ex –smokers should have a KCO% predicted >65%

Exclusion Criteria

Participants with any of the following will not be included in the study

- FEV_1<0.5 L
- Current smoking
- Treatment with any macrolide in the preceding month
- Treatment with tetracycline in the preceding month
- Treatment with any oral corticosteroid in month prior to study
- Change in symptom preventer or controller medication in the previous month
- Hypersensitivity to macrolides
- Taking antacid treatment
- Other respiratory diseases including a primary diagnosis of bronchiectasis, pulmonary fibrosis, pulmonary vascular disease or lung cancer
- Inability to attend study visits

- Respiratory tract infection during month prior to randomisation
- Respiratory exacerbation during month prior to randomisation
- Impaired liver function at Visit 2 as shown by AST, ALT, alkaline phosphatase or total bilirubin greater than the 2 times upper limit of normal

Study Population

Participants will be recruited from the Respiratory clinic at John Hunter Hospital. Participants must have a diagnosis of neutrophilic airway disease.

Study Medication

Azithromycin (Zithromax) and identical placebo tablets will be prepared by a compounding pharmacy and delivered to the Pharmacy department of the John Hunter Hospital.

Packaging, Storage, Dispensing and Accountability

The Pharmacy Department of the John Hunter Hospital will be responsible for the packaging of study medication. Tablets will be packaged in glass bottles (brown) that will be labeled with the study name and the instructions for the participant. The medication will be stored securely in the Pharmacy Department and will be dispensed on presentation of a hospital script according to the randomisation schedule.

Patient Allocation

Patients will be randomly allocated to either receive placebo or AZM 250mg daily. They will take the medication for twelve weeks. Both the participant and the investigators will be blinded to the intervention until the conclusion of the trial. The placebo and agent will be packaged similarly and have identical taste. The use of other medications will be assessed at the commencement of the trial and updated with each contact. The patient will be asked about all new medications commenced and changes in the use of regular medications. If a participant commences treatment with other antibiotic agents during the study they will be withdrawn from the study. To aid compliance medication will be dispensed at each visit. Participants will also be questioned about compliance during the regular phone review.

Treatment will be randomised using random permuted blocks of 6. The data manager in the respiratory research unit using the Randomisation Generator Software (11) will generate the randomisation allocations. The randomisation tables for each stratification list will be

provided to the pharmacy department who will dispense active or placebo medication according to the schedule. The investigator and research team working with the participants will not have access to the randomisation schedule during the study.

Side Effects of AZM

At the recommended doses for nonmycobacterial infections AZM has been generally well tolerated in clinical trials with less than 1% of reactions being described as severe. The most common side effects (1-10%) were diarrhoea, abdominal pain, nausea and vomiting. Metabolic side effects including increases in ALT, AST and alkaline phosphatase were noted in some participants (MIMS). No studies have been carried out on pregnant women, but animal studies suggest that azithromycin is not fetotoxic or teratogenic at doses of up to 200mg/kg/day.

Electrolytes and liver function tests (LFTs) will be done at the initial visit and at each monthly visit during treatment. A rise in the electrolytes or LFTs will result in the test being repeated in one week, if the abnormality worsens the drug will be ceased. If the change is stable a clinical decision will be made to either continue or cease the medication. A record of potential symptoms will be recorded during the regular phone interview.

Evaluation of the Study

The primary outcomes will be:

- Decrease in sputum IL-8

Secondary outcomes will be:

- Decreased in airway bacterial load
- Decreased sputum neutrophils
- Decrease in bronchial wall thickening on chest CT scan
- Number of exacerbations during follow-up

Study Schedule (Appendix I)

Run-in period (Visit 1)

Informed consent will be obtained. Participants will be assigned a screening number and their inflammatory subtype determined using induced sputum. A clinical history will be taken that will include the documentation of medication use and an allergy history. Immediate skin

prick testing will be conducted to a range of allergens in participants where this has not been done in the past 12 months. Spirometry will be performed. Sputum will be induced and analysis performed for total cell count and differential. Participants will then enter a 2-week run in phase, during which they will record respiratory symptoms and medication use in a diary (Appendix II). Ex-smokers will be need to have a carbon monoxide diffusion breathing test if one has not been performed in the previous 2 years.

Randomisation Visit (Visit 2)

At the conclusion of the run-in period (2 weeks) participants will attend for repeat sputum induction and clinical assessment. Stable participants with neutrophilic airway disease will then be randomised to receive either azithromycin 250mg daily or placebo; both the investigator and participants will be blinded. This will be continued for 12 weeks. Participants will be contacted two weeks after each visit by phone and their respiratory symptoms assessed, medications reviewed, and potential side effects recorded. Electrolytes and liver function tests will be done at visit 2, and then at each visit. Sputum will be collected for a total and differential cell count and bacterial culture. Blood will be collected and stored for assessment of systemic inflammation and inflammatory gene profiling. A Full blood count, electrolytes and liver function tests will be done at visit 2, and then at each visit. A high resolution CT scan of the chest will be formed in a sub-group of participants to assess bronchial wall thickening.

Treatment Visits (Visit 3 -5)

The participants will be reviewed after each 4 week period of treatment. Symptom scores, spirometry, sputum induction and analysis and blood parameters will be repeated. At the end of the 12-week treatment phase (visit 4) the medication will be ceased.

End of Treatment Visit (Visit 6)

Symptom scores and quality of life scores will be reassessed. Spirometry, sputum analysis and blood parameters will be repeated. A high resolution CT scan of the chest will be formed to assess bronchial wall thickening.

Post Treatment Follow Up (Exacerbation visits and telephone consultations)

After the post treatment visit, participants will be contacted by telephone at least monthly and asked about their symptoms and medication use.

Clinical History and Symptom scores

A standardised clinical history for respiratory symptoms will be taken. The medications the patient is currently taking will also be recorded. Quality of life scores will be assessed using the St George Respiratory questionnaire. Participants will also be questioned about potential side effects of azithromycin (AZM). This will be done on the second visit, when they are randomised to either placebo or AZM, then every visit thereafter and by phone every two weeks between visits. If during the phone contact there are concerns regarding potential toxicity the participant will be reviewed as soon, as is practicable. Individual symptoms will be recorded using visual analogue scales.

Pulmonary Function Tests and Sputum Induction

A sputum induction will be performed as described elsewhere (12). The tests will use a KoKo K313100 spirometer (PDS Instrumentation, Inc.Louisville CO USA). Hypertonic saline (4.5% saline) will be nebulised using a De-Vilbiss ultrasonic nebuliser (Somerset, PA USA) and aerosol delivered via a one-way non-rebreathing Hans Rudolph valve box. Between testing the participant will be asked to expectorate sputum. Each participant will undergo a maximum of 16 minutes nebulisation time and nebulisation time within participants will be kept constant.

Sputum Analysis

Sputum will be analysed as described elsewhere (12). It will be processed within 1 hour of collection. Muco-cellular clumps will be selected from the saliva. A total cell count and differential will be performed. Cytospin slides will be prepared for a differential cell count. Cytokine analysis on sputum supernatant will be performed with ELISA kits to detect IL-8 and NE. Measurements will be made with commercially available immunoassays according to manufacturers recommended protocols.

REFERENCES

1. Simpson JL, Grissell TV, Douwes J, Scott RJ, Boyle MJ, and Gibson PG. 2005. Innate Immune activation in neutrophilic asthma. *Respirology* 10:A17.
2. Gibson PG, Wark PAB, Simpson JL, Meldrum C, Meldrum S, Saltos N, and Boyle M. 2003. Induced Sputum IL-8 gene expression, neutrophil influx and MMP-9 in allergic bronchopulmonary aspergillosis. *Eur Resp J* 21:582-8.
3. Simpson JL, R. Scott, M. Boyle, and P. Gibson. 2006. Inflammatory subtypes in asthma: assessment and identification using induced sputum. *Respirology* 11:54-61.
4. Fujita K, Shimizu T, Majima Y, and Sakakura Y. 2000. Effects of macrolides on interleikin-8 secretion from human nasal epithelial cells. *Eur Arch Otorhinolaryngol* 257:199-204.
5. Yamaryo T, Oishi K, Yoshimine H, Tsuchiashi Y, Matsushima K, and Nagatake T. 2003. Fourteen-member macrolides promote the phosphatidylserine receptor-dependent phagocytosis of apoptotic neutrophils by alveolar macrophages. *Antimicrob Agents Chemo* 47:48-53.
6. Sakito O, Kadota J, Kohno S, Abe K, Shirai R, and Hara K. 1996. Interleukin 1 beta, tumor necrosis factor alpha, and interleukin8 in bronchoalveolar lavage fluid of patients with diffuse panbronchiolitis: a potential mechanism of macrolide therapy. *Respiration* 63:42-8.
7. Yamada T, Fujieda S, Mori S, Yamamoto H, and Saito H. 2000. Macrolide treatment decreased the size of nasal polyps and IL-8 levels in nasal lavage. *Am J Rhinol* 14:143-48.
8. Wolter J, Seeney S, Bell S, Bowler S, Masel P, and McCormack J. 2002. Effect of long term treatment with azithromycin on disease parameters in cystic fibrosis: a randomised trial. *Thorax* 57:212-216.
9. Simpson JL, Powell H, Boyle MJ, Scott RJ, and Gibson PG. 2007. Anti-inflammatory Effects of Clarithromycin in Refractory Non-Eosinophilic Asthma. *Proc Am Thor Society*.
10. Basyigit I, Yildiz F, Ozkara SK, Yildirim E, Boyaci H, and Ilgazli A. 2004. The effect of clarithromycin on inflammatory markers in Chronic Obstructive Pulmonary Disease: Preliminary Data. *Ann Pharmacother* 38:1400-1405.
11. Goddard J. Randomisation Generator. 1 ed, Southampton.
12. Gibson PG, Wlodarczyk JW, Hensley MJ, Gleeson M, Henry RL, Cripps AR, and Clancy RL. 1998. Epidemiological association of airway inflammation with asthma symptoms and airway hyperresponsiveness in childhood. *Am J Resp Crit Care Med* 158:36-41.

Appendix I: Study Schedule

	**V1**	**V2**	**PC-2**	**V3**	**PC-3**	**V4**	**PC-4**	**V5**	**PC-5**	**V6**
**Week**	**-2**	**0**	**2**	**4**	**6**	**8**	**10**	**12**	**14**	**16**
Consent	X									
SPT	X									
History	X									
Medications	X	X	X	X	X	X	X	X	X	X
QOL		X		X		X		X		X
Chest CT Scan		X						X		
Spirometry	X	X		X		X		X		X
Sputum Induction	X	X		X		X		X		X
Sputum Cell Count	X	X		X		X		X		X
Sputum Mediators		X		X		X		X		X
Bacterial Culture		X		X		X		X		X
Blood Safety		X		X		X		X		
Side Effects Check		X	X	X	X	X	X	X	X	
Administer Diary	D1	D2		D3		D4				
Check Diary		X	X	X	X	X	X	X		
Collect Diary		D1		D2		D3		D4		
Study Medication		X		X		X				
Check Study Medication			X	X	X	X	X	X		
Adverse Events		X	X	X	X	X	X	X	X	

Appendix II: Diary Card

**SYMPTOM SEVERITY SCORE:**		**0 = absent, none at all 2 = Moderate Discomfort**																	
		**1 = Mild Discomfort 3 = Severe Discomfort**																	
	NAME:		Example		Week Commencing								Week Commencing						
**1. During the previous 24hrs**			M	T	M	T	W	T	F	S	S		M	T	W	T	F	S	S
	Cough (0-3)																		
	Wheeze (0-3)																		
	Shortness of Breath (0-3)																		
	Times woken last night due to chest ?																		
	Where your activities limited today due to chest ?																		
	Number of puffs of Reliever Medication?																		
	Did you take your Study Medication (Y/N)																		
**COMMENTS:**____________________																			
If you have any further questions about the study or the diary please phone																			
**Jodie Simpson 49214965**																			
